# Study protocol for a controlled trial of an eHealth system utilising patient reported outcome measures for personalised treatment and care: PROMPT-Care 2.0

**DOI:** 10.1186/s12885-018-4729-3

**Published:** 2018-08-23

**Authors:** Afaf Girgis, Ivana Durcinoska, Martha Gerges, Nasreen Kaadan, Anthony Arnold, Joseph Descallar, Geoff P. Delaney, on behalf of the PROMPT-Care Program Group, Sandra Avery, Martin Carolan, Stephen Della-Fiorentina, Kenneth Masters, Andrew Miller, Weng Ng, Tiffany Sandell, Thomas T. Tran, Janelle V. Levesque

**Affiliations:** 1 Centre for Oncology Education and Research Translation (CONCERT) Ingham Institute for Applied Medical Research, Sydney, NSW Australia; 2 South Western Sydney Clinical School, The University of New South Wales, Sydney, Australia; 3 Liverpool Cancer Therapy Centre, Liverpool Hospital, Liverpool, NSW Australia; 4 Illawarra Cancer Care Centre, Wollongong Hospital, Wollongong, NSW Australia; 5 Department of Statistics, Macquarie University, Sydney, NSW Australia

**Keywords:** Patient-reported outcomes (PROs), eHealth, Patient-centred care, Electronic health record, Self-management, Risk-stratified care, Screening, Non-randomized control trial, Supportive care, Survivorship care

## Abstract

**Background:**

Routine assessment and clinical utilisation of patient-reported outcome (PRO) measures can lead to improved patient outcomes. The PROMPT-Care eHealth system facilitates PRO data capture from cancer patients, data linkage and retrieval to support clinical decisions, patient self-management, and shared care. Pilot testing demonstrated acceptability and feasibility of PROMPT-Care Version 1.0. This study aims to implement PROMPT-Care Version 2.0 and determine its efficacy in reducing emergency department (ED) presentations, and improving chemotherapy delivery and health service referrals, compared to usual care.

**Methods:**

Groups eligible to participate in the intervention arm of this controlled trial are patients receiving cancer care (including follow-up). PROMPT-Care patients will complete monthly assessments (distress, symptoms, unmet needs) until voluntary withdrawal or death. In Version 1.0, the care team accessed patients’ clinical feedback reports in ‘real time’ to guide their care, and patients received links to support their self-management, tailored to their PRO responses. Version 2.0 was extended to include: i) an additional alert system notifying the care team of ongoing unresolved clinical issues, ii) patient self-management resources, and iii) an auto-populated Treatment Summary and Survivorship Care Plan (SCP). The control population will be patients extracted from hospital databases of the general cancer patient population who were seen at the participating cancer therapy centres during the study period, with a ratio of 1:4 of intervention to control patients.

A minimum sample size of 1760 (352 intervention and 1408 control) patients will detect a 14% reduction in the number of ED presentations (primary outcome) in the PROMPT-Care group compared with the control group. Intervention patients will provide feedback on system usability and value of the self-management materials; oncology staff will provide feedback on usefulness of PROMPT-Care reports, response to clinical alerts, impact on routine care, and usefulness of the SCPs; and GPs will provide feedback on the usefulness of the SCPs and attitudes towards shared-care models of survivorship care planning.

**Discussion:**

This study will inform the PROMPT-Care system’s impact on healthcare utilisation and utility as an alternative model for ongoing supportive care.

**Trial registration:**

Australian New Zealand Clinical Trials Registry (ACTRN12616000615482) on 12th May 2016 (www.anzctr.org.au).

## Background

Patient-reported outcomes (PROs) clearly place the patient’s voice at the forefront of health care delivery [1], with systems to routinely collect and utilise PROs in clinical settings demonstrated to be feasible and acceptable [2–5]. Routinely screening for symptoms and other PROs and utilising these data to inform patient care has also been demonstrated to lead to significant improvements in patient outcomes and care indicators. In particular, reductions in emergency department visits [6, 7], longer tolerability of chemotherapy [6], improvements in both short- and long-term survival [6, 8], improved health related quality of life [9] and improved communication between patients and clinicians [9–11] have been documented in oncology settings. PROs have also been effectively used in non-oncology settings, including to inform surgical decisions in the orthopaedic setting [12, 13].

We have previously reported the development and acceptability and feasibility testing of an integrated PRO eHealth system, PROMPT-Care (**P**atient **R**eported **O**utcome **M**easures for **P**ersonalised **T**reatment and **Care**) [14, 15]. This system supports routine collection and analysis of cancer patients’ PROs, real-time feedback of PRO results to their cancer care team to inform patient-centred care, and delivers evidence based self-management information to address patient reported problems. Our feasibility study demonstrated that the PROMPT-Care eHealth system is acceptable to the users, i.e. to the patients and cancer care team, and potentially feasible to implement in cancer centres [15]. Integration of the PRO measures into the hospital’s point-of-care oncology information system (OIS), a key feature distinguishing PROMPT-Care from previous oncology-based eHealth systems, was hypothesised to enhance their relevance and usefulness in informing routine cancer care [16]. Our previous testing was not designed to inform the utility elements of the PROMPT-Care system or its efficacy. Therefore, this will be the primary purpose of the proposed study.

We have used the term *patient* in reference to all people diagnosed with cancer who are currently on treatment and in follow-up.

### Objective

The overall objective of this study is to implement the PROMPT-Care 2.0 eHealth platform and determine its efficacy among cancer patients at four tertiary hospitals. Specifically, this study will test whether web-based routine collection of PROs, combined with automated alerts to clinical teams and provision of patient self-management resources, result in reduced emergency department presentations, and improved chemotherapy delivery and health service referrals. The study will also evaluate system utility and potential benefits and barriers to PROMPT-Care implementation in routine care from both the patient and healthcare professional perspective.

## Methods/design

### Setting

The study is being undertaken in the cancer therapy centres of four participating hospitals, with oversight of the implementation undertaken by a clinical study lead: Liverpool Cancer Therapy Centre and Macarthur Cancer Therapy Centre (GD), Illawarra Cancer Care Centre and Shoalhaven Cancer Care Centre (AA).

### Ethics approval

Ethics approval was obtained from the Human Research Ethics Committees of South Western Sydney, and Illawarra Shoalhaven Local Health Districts (Reference No. HREC/15/LPOOL/287).

### PROMPT-care intervention

As previously reported [14, 15], the PROMPT-Care platform facilitates patients completing PRO measures online through standardised assessment tools using an electronic device (e.g. tablet, iPad, smart phone, or computer) and automatically converts these data into a format (HL7 messages) [17] that is transferred directly into the patient’s point-of-care OIS in ‘real time’, following an automated matching verification process to ensure the correct record is populated. The point of care system used in this trial was Mosaiq ™ (Elekta Medical Systems, Sunnyvale, CA). PROs assessed include: Distress Thermometer (DT) and associated checklist [18], the Edmonton Symptom Assessment Scale (ESAS) [19], and the Supportive Care Needs Survey-Screening Tool 9 (SCNS-ST9) [20].

Feedback received during feasibility testing of pilot configuration for the PROMPT-Care system (Version 1.0) [15], highlighted additional patient and clinical team needs. As a result, Version 2.0 of the PROMPT-Care system has been extended to include the following elements: i) an additional alert system notifying the cancer care team of patients with ongoing unresolved issues, ii) tiered patient self-management resources, and iii) an auto-populated Treatment Summary and Survivorship Care Plan (SCP).

**Fig. 1:**
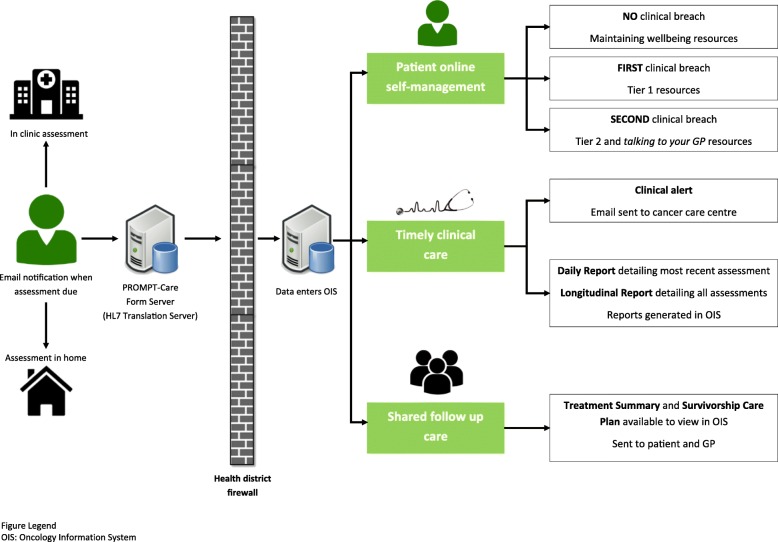
PROMPT-Care 2.0 system overview

**Fig. 2:**
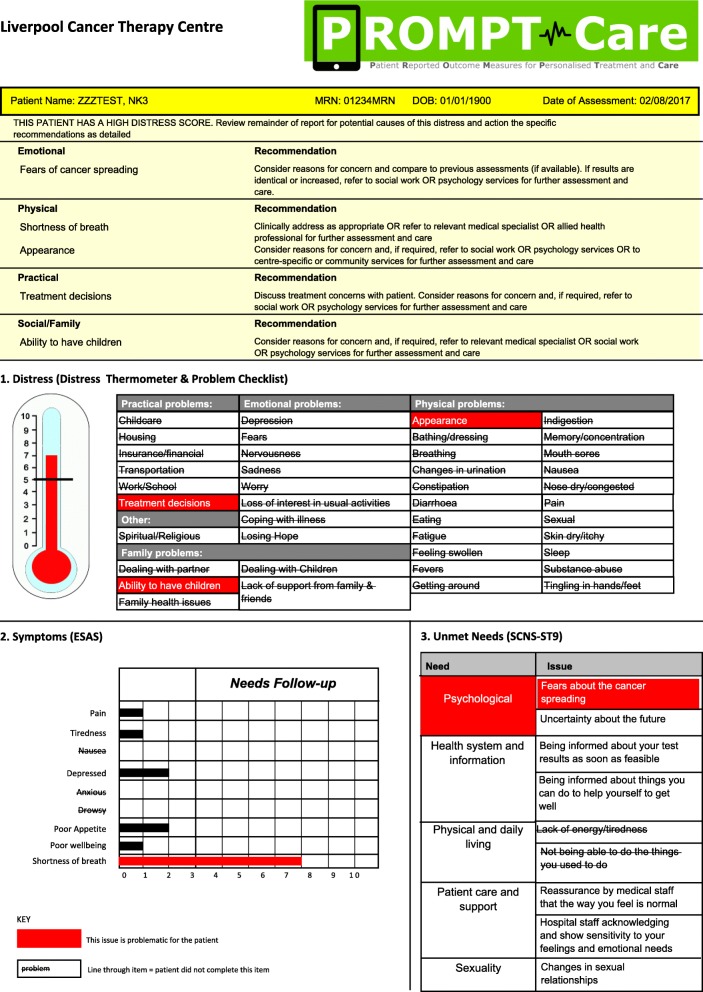
Sample clinical feedback report

Version 2.0 of the PROMPT-Care intervention consists of three components (Fig. 1.):

1. Timely clinical care
  Clinical feedback reports
  Using previously reported algorithms [21], the uploaded PRO data is presented in a clinical feedback report (Fig. 2), which includes a basic one-page summary of the results of the most recent assessment, recommended clinical actions and suggested referrals, as well as a longitudinal report (Fig. 3) of patients’ scores over time on the PRO scales. The reports are available ‘real time’ for clinical staff to review in the clinic with patients.

**Fig. 3:**
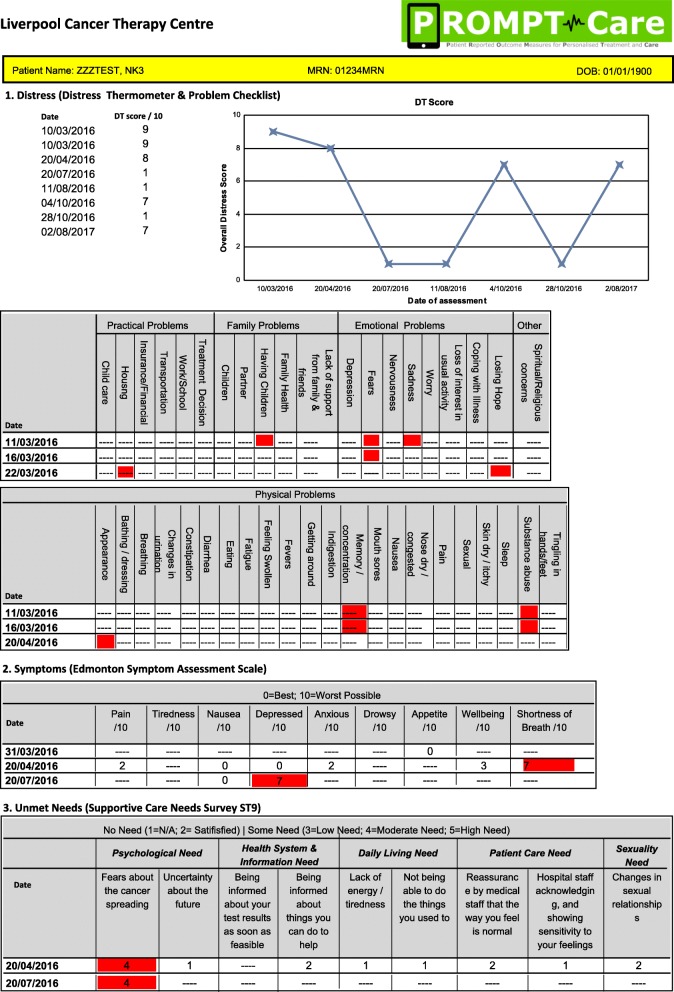
Sample longitudinal feedback report
  Clinical alerts
  If the patient’s scores on any of the PROs breach a predefined threshold on two consecutive assessments, an automated alert will be generated in the OIS, with an email received by a designated member of the cancer centre team, who will review the PROMPT-Care report and follow the care pathway agreed for that cancer centre.
2. Tiered patient self-management

**Fig. 4:**
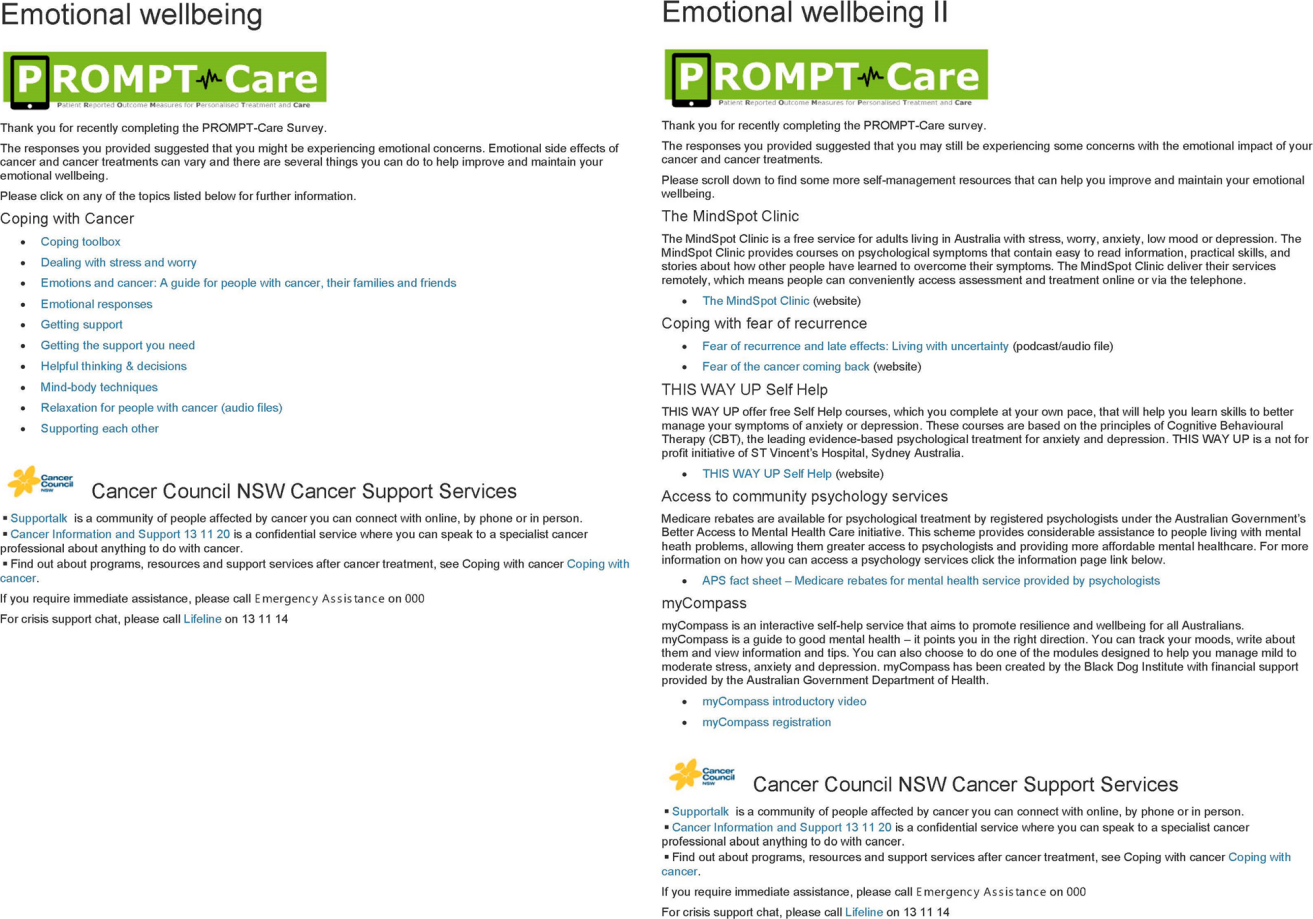
Screenshot of patient self-management Tier I and Tier II pages (emotional well-being)

Upon completing their PROMPT-Care assessment, patients will receive an email which directs them to a website containing tailored self-management information resources (Fig. 4) to address issues of concern they identified in their assessment. In response to patient feedback in the feasibility study [15], the PROMPT-Care system was modified to provide patients with a tiered approach to their self-management support. The first time a patient breaches a PRO item, they will receive a link to generic information resources via one of the four distinct domain-specific webpages hosted on the Cancer Institute NSW eviQ website: emotional well-being, physical well-being, social/family well-being, practical problems. Patients who do not breach any items will receive a link to the “maintaining well-being” page, to support their continued general health. If a patient breaches the same item on two consecutive PROMPT-Care assessments, they will receive a link to more dynamic and interactive resources such as videos, podcasts, or interactive self-help programs (where available), as well as links to resources to facilitate effective communication with their GP and appointment preparation.

3. Shared follow up care

For patients who complete their primary treatment (chemotherapy, radiotherapy) and transition to follow-up care during the trial, an auto-populated Treatment Summary will be generated within the OIS, approved by the treating clinicians, and then sent to both the patient and their nominated GP. The treatment summary will contain information regarding the patient’s diagnosis, treatments received, complications, ongoing medications, and support services to which the patient was referred. For patients diagnosed with colorectal, prostate, breast or gynaecological cancers, an accompanying SCP, summarising potential long-term effects of treatment, recommended tests and follow-up appointments will also be sent.

### Study population

#### Patients

Eligible patients are people who are either currently receiving cancer care (including follow-up care) or have recently been diagnosed with cancer and are scheduled to commence cancer treatment at one of the four participating sites. Eligibility criteria include a confirmed diagnosis of cancer, age 18 years or over, cognitively able to provide informed consent and understand the surveys, and ability to complete the survey in English. Exclusion criteria are having a diagnosis of a blood cancer and not having access to the Internet outside of the clinic.

#### Oncology staff

All staff who provide care in the oncology departments at the participating hospitals are eligible to participate.

#### General practitioners (GPs)

All GPs nominated by a participating patient as their primary care provider will be eligible to participate.

### Control population

A list of potential control individuals (minimum *n* = 1408) will be extracted from hospital databases of the general cancer patient population who were seen at the participating cancer therapy centres during the study period.

### Procedure

#### Oncology staff engagement and consent

Oncology staff (including specialists and nurses) will be invited to participate via email and sent an introductory summary, information sheet and consent form. Treating clinicians are asked to provide permission for research staff to contact their patients, and consent to participate in an evaluation interview at study close. Consenting oncology staff will receive training resources and participate in orientation sessions on how to use the PROMPT-Care system in routine clinical practice at study start along with refresher resources and orientation throughout the trial as needed.

#### Patient identification and consent

Participating clinicians will review their clinic lists to identify eligible patients who have appointments scheduled within the coming two (2) months. Two patient recruitment approaches will be utilised to achieve a similar proportion of both patients in active treatment and those in follow-up care.

##### In clinic

Eligible patients attending a cancer care clinic will be invited to participate by a member of the clinical trial team in the waiting area. The trial research member will explain the study in detail and provide patients with a study pack containing a letter of invitation, participant information and consent form, demographics survey and reply-paid envelope. Consenting patients will be asked to complete a consent form and their first PROMPT-Care assessment just prior to their appointment.

##### Mail-out

For patients who require additional time to consider participation, or those in follow-up who do not have regular clinic appointments, research staff will mail out a study pack inviting them to participate, then follow up by phone to answer any questions and provide additional information, as required.

#### Assessment completion

Patients will complete PROMPT-Care assessments on a monthly basis and will be followed up for a minimum of four months. Patients will have the option to complete assessments either at the hospital in clinic, their home or any public community location. Patients completing assessments in clinic will be provided with a tablet device in the waiting area just prior to their scheduled appointment. Patients who complete assessments from home or in community locations will receive an email containing the survey link three days before their assessment is due.

### Measures

#### Outcomes

The primary outcome is number of emergency department (ED) presentations observed during the study period. Details about ED presentation dates, reason for presentation and any resulting admissions will be extracted from the electronic medical record (EMR) (Table 1).

**Table 1:** Primary and secondary outcome data collected

Data	Description of data	Source of data
Primary Outcomes		
Emergency Department (ED) presentations	• ED presentations (date, number of visits) • Length of stay • Reason for presentation	Extracted from electronic medical record (EMR)
Secondary Outcomes		
Time on chemotherapy	• Planned chemotherapy regimen • Actual regimen start and end date • Toxicities, changes to treatment delivery and reasons	Extracted from oncology information system (OIS)
Referral to allied health services	• Date of referral and allied health service type • Reason for referral eg. emotional distress, case management etc.	Extracted from EMR & OIS

The secondary outcomes are time receiving active chemotherapy and referrals to allied health services. Details of planned and actual chemotherapy regimens as well as any toxicities and changes to treatment delivery will be extracted from the OIS. Date, reason and number of referrals to allied health services will be tabulated and extracted from the medical record and OIS.

#### Patient clinical and socio-demographic characteristics

Upon consenting, participants will complete a questionnaire about socio-demographics including: marital status, education level, employment status and language spoken at home. Additional demographics (eg. age, gender and need for interpreter) and clinical characteristics such as date of diagnosis, site (ICD-10), stage (TNM classification) and treatment details will be extracted from the OIS.

#### System utility evaluation

##### Patient evaluation

Patients will complete periodic online progress evaluation surveys following the completion of their third, sixth and ninth PROMPT-Care assessments. Patients will be asked about the usability of the system, preferences for timing of completing PROMPT-Care assessments, satisfaction and usefulness of the system, suitability and value of the self-management materials, and suggestions for further refinement. A sub-set of patients (approximately 10–20 patients) will also be invited to participate in semi-structured interviews at study completion in order to further explore themes identified in the evaluation surveys.

##### Healthcare professional evaluation

Participating oncology staff and GPs will be invited to participate in evaluation surveys and semi-structured interviews at study completion. Oncology staff will be asked questions about, how they used the PROMPT-Care reports in clinical practice and their usefulness, their response to the clinical alerts, how PROMPT-Care impacted routine care, and their views on the Treatment Summary and SCPs. GP data will be analysed to evaluate the content and suitability of the Treatment Summary and SCPs. It will also be used to gauge attitudes towards shared-care models of survivorship care planning.

##### System usage statistics

Data on the use of the PROMPT-Care system will be extracted from the OIS and evaluated to inform: frequency of report usage, clinical alert activity, assessment data transfer, and IT system functioning. User and technical system errors will also be monitored by research staff and recorded in an error log of IT issues and associated resolutions e.g. firewall upgrades, server downtime, participant report of IT problems completing assessments or accessing resource webpages. Patient interaction with and use of the self-management resources will be analysed by Google Analytics [22] and ClickMeter [23] over time. Google analytics will be used to gather data on the number of users and views of the domain-specific resource webpages (eg. emotional, physical, social/family, maintaining well-being, and practical problems), whereas ClickMeter will be used to track clicks into the individual resources (*n* = 114), sitting within each domain page. System usage data will be summarised using simple descriptive statistics and will be presented as counts, mean scores, standard deviations and percentages.

### Sample size

A minimum sample size of 1760 (352 intervention and 1408 control) patients is required to detect a 14% reduction in the number of ED presentations in the PROMPT-Care group compared with the control group. This is based on the assumed rate of ED presentations being 1.4 visits per patient during the study period, a 1:4 ratio of PROMPT-Care to control group patients, 80% power and 5% statistical significance.

### Statistical analyses

Descriptive statistics will be generated for all socio-demographic and clinical characteristics, and outcome measures. A multivariable Poisson or negative binomial regression (depending on over-dispersion) will be used to determine whether the rates of ED presentations were different between the PROMPT-Care and control groups adjusting for covariates (such as age, sex, stage of disease, and treatment status). Number of referrals to allied health services will be analysed similarly. Multivariable Cox proportional hazards model will be used to analyse length of time from start to end of chemotherapy adjusting for covariates.

### Qualitative analysis

Interviews with patients and health professionals will be audio-recorded, transcribed verbatim, and analysed using thematic analysis [24]. Two researchers will independently read the transcripts and generate initial codes. Identified codes will then be collated into emerging themes. Themes will then be refined, with discrepancies resolved through discussion and consensus.

## Discussion

To date, the impact of collecting and utilising PROs in the oncology setting have been studies in defined groups of patients. The results from this study will contribute important new evidence to the literature, with its inclusion of a broad population of patients who are currently undergoing cancer treatment or are in follow-up, and patients with a wide range of tumour types. PROMPT-Care Version 1.0 has previously been demonstrated to be feasible and acceptable [14, 15]. This project will provide evidence regarding the impact of the expanded and improved PROMPT-Care Version 2.0 system on healthcare utilisation, including emergency department presentations, chemotherapy adherence and referral to allied health services; the acceptability of the tailored, stepped self-management resources; and usefulness of newly introduced strategies to facilitate shared follow-up care - the Treatment Summary and SCPs.

This information will be used to guide further revisions of the PROMPT-Care system and aid its wider implementation in other cancer centres in Australia; and inform its potential as an alternative model of providing ongoing patient supportive care. The resulting eHealth platform will be an evidence-informed tool which supports and enables cancer patients to achieve and maintain improved well-being and better cancer outcomes.

## Abbreviations

CINSW: Cancer Institute NSW
DT: Distress Thermometer
ED: Emergency Department
EMR: Electronic Medical Record
ESAS: Edmonton Symptom Assessment Scale
GP: General Practitioner
MRN: Medical Record Number
OIS: Oncology Information System
PRO: Patient Reported Outcome
PROMPT-Care: Patient Reported Outcome Measures for Personalised Treatment and Care
SCNS-ST9: Supportive Care Needs Survey – Screening Tool 9 items
SCP: Survivorship Care Plan
TNM: Tumour, node and metastases
